# Case of Uterine Hemorrhage, in Which the Operation of Transfusion Was Successfully Performed

**Published:** 1825-10

**Authors:** C. Waller


ART. II.—*Case of Uterine Hemorrhage, in which the Operation of Transfusion was successfully performed.* By C. WALLER, Esq.

I HAVE for a long time been of the opinion, that the transfusion of blood from the vessels of one person into those of another, is an operation that ought by no means to be ridiculed, much less to meet with that hasty condemnation which it has been its lot to experience from some of the members of our profession. This opinion has been further strengthened by an attentive perusal of the experiments on the dog, which have been so ably conducted, and so accurately described, by Dr. Blundell. I have also had some conversation with the Doctor on the subject; and the result has been to produce a firm conviction on my mind, that in cases (for example) of profuse uterine hemorrhage, if, notwithstanding the cessation of the bleeding, and the employment of the usual means, we find that the alarming symptoms of collapse increase or continue, so that there is reason to presume that, in the course of a few hours, the patient will die, it becomes our bounden duty not to neglect the trial of this operation. It is not proposed, till we have a greater accumulation of evidence on the subject, to employ the remedy *at once* in every case of alarming hemorrhage, but in those cases only where the usual means have been tried and failed: and, surely, there can be nothing ridiculous in this, unless we are so enveloped and shrouded in our own prejudices and opinions, that we are determined to consider every thing ridiculous which is of a novel nature.

The female, whose case I am about to narrate, was of a very delicate habit and strumous appearance. She was a patient in the charity of which I have the superintendence, and was attended by my friend, Mr. Jesse, of Manchester. I had myself seen her about two o'clock in the afternoon, in consequence of Mr. J.'s not being in the way. A large quantity of liquor amnii had escaped just before I arrived at the house. On examination, the head was still above brim, the os uteri nearly dilated, and, as well as the external parts, very dilatable. The pains, however, declined, and I left her, desiring her to send again when they began to increase: this took place in about three hours afterwards, and the child was soon born. Shortly after this, I was requested to go to Mr. Jesse's assistance; and, when I entered the room, the patient was to all external appearance dead: she was lying on her back, completely blanched, her face pale as the sheet that covered her; there was not the slightest tinge of redness on the lips; her hands and feet cold; the power of deglutition suspended; and there was no apparent respiration. On placing my finger upon the wrist, I could just discover the pulsation of the artery, with, however, occasional intermissions.

On inquiry, I found that the expulsion of the placenta, which was accomplished by nature, was followed by profuse hemorrhage, and that the symptoms of collapse had instantly supervened. The appropriate remedies—viz. the application of cold and frictions,—were immediately had recourse to; and, on my arrival, as the uterus was still large, although there was a considerable degree of contraction (for it felt hard), and as there was a slight draining of blood, I continued the cold applications to the belly, and applied warmth to the feet; her lips were also smeared with brandy. After a short time she was able to swallow, and then brandy and ammonia were liberally given. For a few seconds after the administration of these stimuli, the pulse would rise a little, but soon sank again. The bleeding soon ceased entirely; and, as she was getting generally cold, we wrapped her up in blankets, and continued the brandy, but with no greater success.

After persevering for about an hour and a half in these measures, (which I think was giving them a fair trial,) and finding that we were gaining no ground, I determined upon consulting Dr. Blundell as to the propriety of performing the operation of transfusion, especially as he had very kindly offered to come to my assistance, in case of such an emergency as the present, whether it occurred in public or in private practice.

The patient resided in the neighbourhood of Finsbury-square; some little time, therefore, necessarily elapsed before we returned, and the impression upon my mind was most decidedly that we should find her dead. Contrary, however, to my expectation, I found her not only alive, but I thought the pulse was rather more perceptible, although it would not bear the pressure of the finger; we therefore agreed to have every thing in readiness for the operation, but not to perform it at present.

After waiting nearly two hours, we found that the pulse certainly was not lower, and for the last hour it appeared to beat with more regularity. Dr. B. and myself then left her for about an hour, and on our return it was evident that the patient had lost ground; the pulse was fallen again, and she was still lying in that alarming state of faintness, that, although death was not indisputably certain, it was the deliberate opinion, both of Dr. Blundell and myself, that the presumption against her surviving was so strong, that it became our duty to act. The operation was, therefore, determined upon.

The vein in the bend of the arm was laid bare, and an incision of sufficient extent to admit the pipe of the syringe was made into it. As some little blood flowed from the vein, it was found necessary to pass a blunt needle under it, a little below the orifice; by this we could completely restrain it. Now, as in these cases the patient has not a tea-spoonful of blood to spare, it will be found advisable, in future operations of this nature, to adopt this plan before the opening into the vein is made.

The syringe used by Dr. B. was similar to the common injecting syringe, and contained two ounces. It is, of course, highly necessary that all air should be excluded, as much mischief might accrue from its injection into the vein. The blood was drawn from the patient's husband into a tumbler, and Dr. B. stood ready with his syringe to absorb it instantly,—in fact, while it was flowing: it was then immediately introduced into the orifice in the vein, and cautiously injected. No effect appeared to be produced by the first injection of two ounces, but towards the end of the second there was an approach to syncope; the pulse fell a little; there was sighing, with efforts to vomit, although nothing was ejected. These symptoms ceased spontaneously in the course of a minute or two: indeed, they were very similar to those which occur after venesection, when there is a tendency to, though not actual deliquium.

I should have stated that the patient threw up the contents of her stomach previous to the commencement of the operation; and, whether this was a repetition of her sickness, or whether it was produced by the injection, future observations must prove. In one of these cases which Dr. Blundell has witnessed (in a female), symptoms somewhat similar were induced; but, in the case of Brazier (a male), there were no such symptoms, although a much larger quantity of blood was injected.* As,

* Vide *Medico-Chir. Transactions*, vol. x.

however, in the present limited state of our experience, great caution is required in conducting the operation, and as the patient expressed herself easy, it was considered advisable to proceed no further. Her pulse, which before the operation was 120, was now 110, but of course still feeble.

On our visit in about six hours afterwards, we were gratified in finding that our patient had rallied considerably: her pulse was now 100, and much rounder. She complained much of hunger: a nourishing, but unstimulating, diet was therefore allowed her: and she recovered without a single unpleasant symptom. She slept well, and awoke refreshed, and never complained of that unpleasant state of head so common after hemorrhage. In fact, she required no medicine at all, except a table-spoonful of castor-oil, which was given her on the fourth day, and which purged her much. This irritable state of bowels, as your readers well know, is a common occurrence in these cases. There was no attempt at union by adhesion, and it was not till after the lapse of many days that the vein was healed.

Now, it may be said that this patient might have recovered if no blood had been injected; and this is an objection which it is impossible peremptorily to deny; but, as stated above, it was our *deliberate* opinion that the chances were formidably against her, and I think in that opinion any gentleman who had seen the case would have concurred. But this kind of objection might be made to every remedy which is now, or ever has been, in use. We have yet to learn in these, as well as in many other cases, what the powers of nature can do; for it is impossible demonstratively to prove whether the desired change has been produced by nature or by the remedy. It by no means follows that, because death did not take place immediately after the irruption of the blood, that therefore the patient would have survived; for there was in this instance nothing like a rally, even after the lapse of six hours. In the only fatal case of hemorrhage which it has been my lot to witness, the patient survived three hours after the discharge had ceased, and then expired, although the usual means were vigorously had recourse to during the whole of that period. I have frequently, very frequently, regretted that transfusion had not been tried in this instance; though, had the patient been saved by the operation, a doubt might have been raised whether the injection of the blood was the cause of recovery.

Again it may be objected, that the injection of so small a quantity as four ounces of blood was not likely to save a woman who was sinking from hemorrhage. To this I would reply, by stating it to be my firm conviction that this quantity, though small, was sufficient to turn the scale in her favour. Be it remembered that the intention of the operation was not to restore the patient to her former vigour, but merely so far to increase the quantity of the circulating fluid as to ensure the continuance of the heart's contractions, till the system could recruit itself by the more circuitous route of digestion and sanguification. I would also appeal to those who have been in the habit of seeing much of uterine hemorrhage, whether they have not occasionally met with cases where the patient has been brought so low, that another gush of blood, though it should not exceed four ounces, would in all probability be sufficient to prevent the patient rallying: if so, it justifies the opinion I have expressed above, that the quantity, though small, was sufficient to turn the scale in her favour.

The above case has, however, proved, with all the force that a solitary fact is capable of proving any thing—

1st. That the operation is exceedingly simple, and can be performed with the greatest ease.

2dly. That four ounces of blood may be injected into the veins of a woman who is in danger of sinking from hemorrhage, without producing any noxious effects; or, at the most, without occasioning more unpleasant symptoms than often occur in consequence of the abstraction of a small quantity of blood in some subjects; and that, therefore,

3dly. It is highly probable that the blood thus injected is at once made subservient to the general purposes of the circulation.

111, *Aldersgate-street; August*, 1825.

